# Association between *MMP-1* g.-1607dupG Polymorphism and Periodontitis Susceptibility: A Meta-Analysis

**DOI:** 10.1371/journal.pone.0059513

**Published:** 2013-03-20

**Authors:** Dandan Li, Qi Cai, Lan Ma, Meilin Wang, Junqing Ma, Weibing Zhang, Yongchu Pan, Lin Wang

**Affiliations:** 1 Institute of Stomatology, Nanjing Medical University, Nanjing, China; 2 Department of Epidemiology, Nanjing Medical University, Nanjing, China; Sanjay Gandhi Medical Institute, India

## Abstract

**Background:**

Matrix metalloproteinase-1 (MMP-1) plays an important role during the destruction of periodontal tissue. Although multiple studies had focused on the association between *MMP-1 g.-1607dupG* and periodontitis susceptibility, the results remained inconclusive. The purpose of this meta-analysis was to explore its role in the development of periodontitis.

**Methods:**

Retrieved studies from Pubmed, Web of Science, Medline and Google Scholar Search regarding *MMP-1 g.-1607dupG* and periodontitis susceptibility were included into the final analysis with definite selection and exclusion criteria. Overall and stratified analyses based on disease type, severity, ethnicity and smoking status were performed. Odds ratio (OR) and 95% confidence interval (CI) were used to evaluate the association between *MMP-1 g.-1607dupG* and periodontitis susceptibility, while Q test and Egger’s test were adopted respectively to assess heterogeneity among studies and publication bias.

**Results:**

A total of 1580 periodontitis cases and 1386 controls in 11 case-control studies were included in the meta-analysis. The pooled results showed significant association between periodontitis susceptibility and *MMP-1 g.-1607dupG* polymorphism in homozygote (2G/2G versus 1G/1G, OR = 1.50, 95% CI = 1.02–2.20) and dominant model analysis (2G/2G+2G/1G versus 1G/1G, OR = 1.28, 95% CI = 1.04–1.57). For subgroups by type of periodontitis, increased risk of chronic periodontitis was observed on heterozygote (2G/1G versus 1G/1G, OR = 2.01, 95% CI = 1.58–2.56) and dominant model (OR = 1.27, 95% CI = 1.03–1.57). Furthermore, similar association was also detected in severe chronic periodontitis (2G/2G versus 1G/1G, OR = 2.15, 95% CI = 1.35–3.43; 2G/2G+2G/1G versus 1G/1G, OR = 1.64, 95% CI = 1.12–2.39; 2G/2G versus 2G/1G+1G/1G, OR = 1.86, 95% CI = 1.31–2.64).

**Conclusions:**

Our meta-analysis demonstrated that *MMP-1 g.-1607dupG* polymorphism was associated with chronic periodontitis, especially the severity of the disease condition.

## Introduction

Periodontitis are of the most common oral diseases around the world with high prevalence of 10%–15% [1], constituted by two major types: chronic periodontitis (CP) and aggressive periodontitis (AgP). As kinds of inflammatory diseases, they could not only cause great periodontium damage by interaction between pathogens challenge and host immunological reaction [2], [3], but also contribute to tooth loosening and loss. Furthermore, their potential adverse effects on systemic health [4], such as adverse pregnancy outcome [5], diabetes mellitus [6], cardiovascular disease [7], [8] and some other general diseases [9], [10], should also be paid much attention to.

Matrix metalloproteinases (MMPs), a series of proteolytic enzymes responsible for the degradation of extracellular matrix and basement membranes in the beginning and developing courses of a wide range of diseases [11]–[13], have been verified to be involved in the pathogenesis of periodontitis [14], [15]. Among them, MMP-1 is the most abundant component of the periodontal tissue matrix [16], [17], regulating the degradation of native interstitial collagens [18]. It is worth noting that fibrillar collagens types I and III, the predominant types of interstitial collagens in periodontium which are resistant to most proteinases, can be degraded by MMP-1 [19]. Consequently, tissue inhibitors of metalloproteinases (TIMPs) have been used as the hypurgia for human periodontitis to control MMP-mediated extracellular matrix breakdown [16].

Owing to the important role of MMP-1 in the pathogenesis of periodontitis, a variety of molecular epidemiological studies have been conducted to explore the association between *MMP-1* polymorphisms and the susceptibility of periodontitis. The guanine addition at the -1607 position, the substitution of guanine for adenine at position -519 as well as the adenine to thymidine mutation at position -442 of the *MMP-1* gene promoter were supposed to be the functional polymorphisms associated with periodontitis. However, polymorphism at position -1607 (*MMP-1 g.-1607dupG*, rs1799750) was the most extensive studied locus. During the past few years, various studies from different ethnic groups were conducted to test its relevance with periodontitis susceptibility. Nevertheless, these results still remained inconsistent, which warranted us to perform a meta-analysis to further clarify its role in the pathogenesis of periodontitis.

## Methods

### Search Strategy and Data Extraction

To systematically retrieve all the case-control studies related to the association between *MMP-1 g.-1607dupG* and periodontitis risks, databases of PubMed, Web of Science, Medline and Google Scholar Search were searched (by May 30, 2012) with the key words “periodontitis”, “*MMP-1*” (or “matrix metalloproteinase-1”) and “polymorphism” (or “variant”). The references of all identified publications were manually searched for additional studies. In the search results, only English articles were taken in. All selected studies complied with the two main criteria: (1) independent case-control study evaluating the association between *MMP-1* polymorphism and periodontitis susceptibility; (2) the number or frequency of genotype given in detail. Studies with insufficient information (e.g. neither the frequency nor the number of genotype was given) were excluded. Two investigators (Li and Pan) independently extracted the data and reached a consensus in order to minimize the bias and improve the reliability. Then information including the first author’s name, year of publication, country of origin, ethnicity, type of periodontitis, source of control, number of cases and health controls, genotyping method, Hardy-Weinberg Equilibrium among controls and the main result of each publication was picked up. Different ethnic descents were classified as Caucasian, Asian or Mixed (derived from an admixture of different ethnic groups).

### Statistical Analysis

Hardy-Weinberg equilibrium of the genotype distributions among controls were estimated by a goodness-of-fit χ^2^ test. The association between *MMP-1 g.-1607dupG* and susceptibility of periodontitis was estimated by odds ratio (OR) and 95% confidence interval (CI). In addition to the overall analysis, stratified analyses based on disease type, severity, ethnicity and smoking status were performed respectively. The 2G/2G+2G/1G versus 1G/1G and 2G/2G versus 2G/1G+1G/1G comparison were estimated to assume dominant and recessive effects of the variant 2G allele respectively. The statistical significance of pooled ORs was determined by Z test. Q test based on *P* and I^2^ value was used to assess heterogeneity among studies. I^2^ was a value that could describe the percentage of variation across studies. The bigger I^2^ value, the stronger heterogeneity is. *P*>0.05 for the Q-test indicated no significant heterogeneity across studies, and the fixed-effects model (the Mantel-Haenszel method) was applied; if not, the random-effects model was used (the DerSimonian and Laird method) [20].

Publication bias was evaluated with the linear regression asymmetry test by Egger et al [21]. *P*<0.05 was used as an indication for the presence of potential publication bias. All analyses were done with STATA software (version 11; StataCorp LP, College Station, TX, USA), and the *P* values were all two-sided.

## Results

### Characteristics of Studies

**Figure 1:**
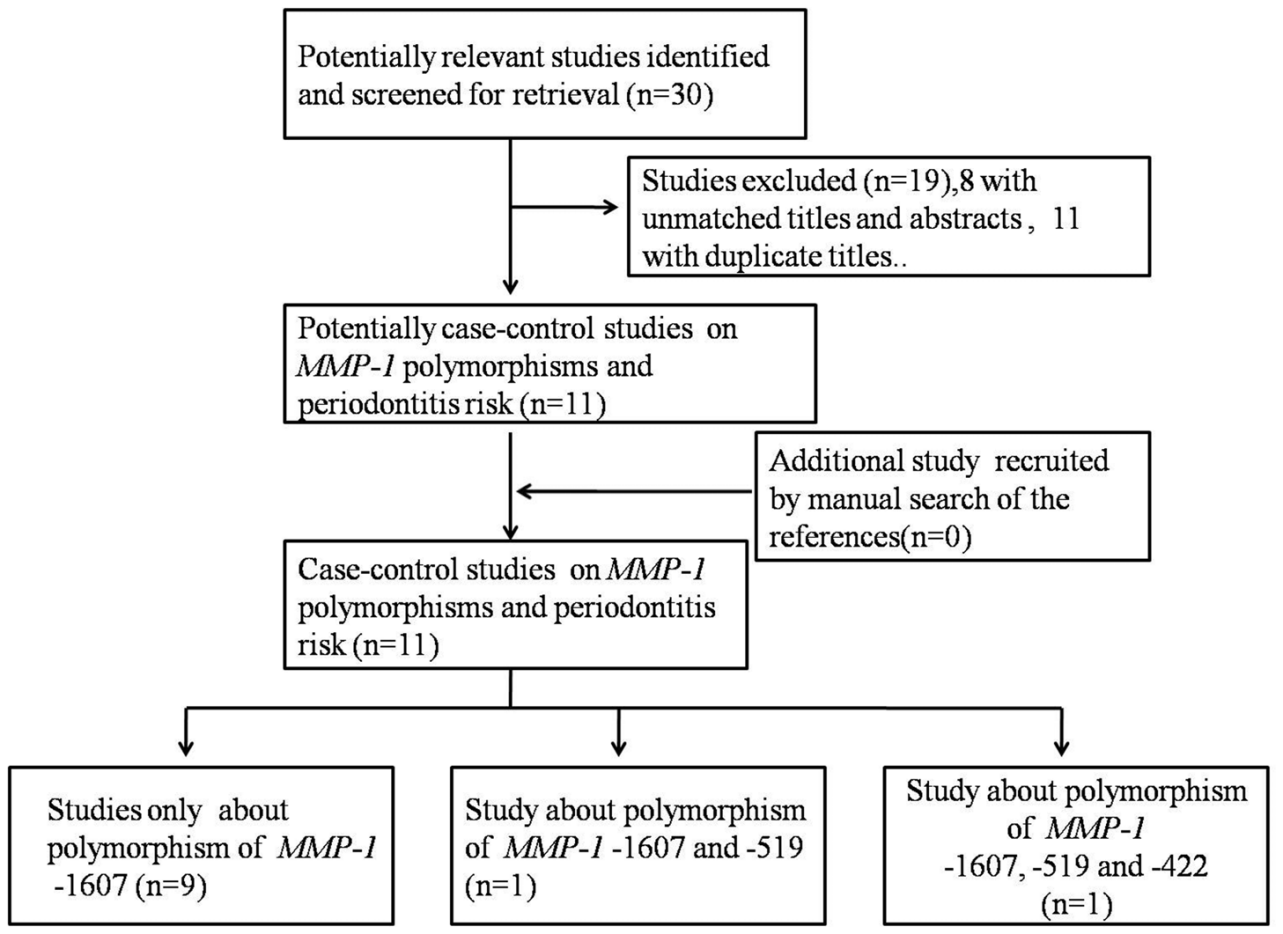
Studies identification diagram.

11 relevant papers with 1580 cases and 1386 controls about *MMP-1 g.-1607dupG* polymorphism were recruited and put into the final meta-analyses [22]–[32] (Figure 1). All studies were case-control studies, including nine studies [22], [23], [26]–[32] for CP, one study [25] for AgP and one [24] for both. There were four studies of Asian descent [24]–[26], [31], four studies of European descent [22], [23], [28], [29] and three studies of mixed ethnicity descent [27], [30], [32]. The distribution of genotypes in the controls was not in agreement with HWE for three studies [28], [30], [31]. (Table 1).

**Table 1:** Relevant literatures concerning relationships between *MMP-1* polymorphisms and periodontitis.

Author	Year	Country	Ethnicity	Type of disease	Source of control	Sample size ofcase/control	genotyping methods	HWE among controls	Result
*MMP1* -1607*dupG*									
de Souza [22]	2003	Brazil	Caucasian	CP	HB	50/37	PCR-RFLP	0.87	+^a^
Holla [23]	2004	Czech	Caucasian	CP	PB	133/196	PCR-RFLP	0.52	+^a^
Itagaki [24]	2004	Japan	Asian	AgP	HB	37/142	Taqman	0.47	−
				CP	HB	205/142	Taqman	0.47	−
Cao [25]	2005	China	Asian	AgP	HB	40/52	PCR-RFLP	0.77	+
Cao [26]	2006	China	Asian	CP	HB	60/50	PCR-RFLP	0.99	+^b^
Astolfi [27]	2006	Brazil	Mixed	CP	PB	114/109	PCR-RFLP	0.68	−
Pirhan [28]	2008	Turkey	Caucasian	CP	HB	101/97	PCR-RFLP	0.009	−
Ustun [29]	2008	Turkey	Caucasian	CP	HB	126/54	PCR-RFLP	0.75	−
Repeke [30]	2009	Brazil	Mix	CP	PB	178/190	PCR-RFLP	0.0005	−
Loo [31]	2011	China	Asian	CP	PB	280/250	PCR-RFLP	1.36E−39	+
Luczyszyn [32]	2012	Brazil	Mix	CP	HB	60/67	PCR-RFLP	0.07	−
*MMP1* -519 A>G									
Holla [23]	2004	Czech	Caucasian	CP	PB	133/196	PCR-RFLP	0.52	−
Pirhan [28]	2008	Turkey	Caucasian	CP	HB	102/97	PCR-RFLP	0.009	−
*MMP1* -422 A>T									
Holla [23]	2004	Czech	Caucasian	CP	PB	133/196	PCR-RFLP	0.52	+^c^

+The significant relevance between *MMP-1* polymorphism and the risk of periodontitis was picked up in this article.

−No association between MMP-1 polymorphism and the risk of periodontitis was picked up in this article.

a only association with severe chronic periodontitis in non-smoking population.

b only association with severe chronic periodontitis.

c only association with severe chronic periodontitis in smoking population.

There were only 2 studies for *MMP-1*-519 A/G, neither found any relationship with periodontitis [23], [28]. Meanwhile, *MMP1* -422 A/T was only mentioned in one literature [23] (Table 1). Therefore, meta-analyses for the latter two SNPs were not performed in the present study.

### Overall Analysis

In general, the *MMP-1* -1607 2G/2G homozygote was significantly associated with an increased risk of periodontitis compared with wild-type homozygote (1G/1G) (OR = 1.50, 95% CI = 1.02–2.20). Significant association was also found in the dominant genetic model (OR = 1.28, 95%CI = 1.04–1.57), but neither in recessive model (OR = 1.31, 95%CI = 0.95–1.79) nor in heterozygote comparison (OR = 1.23, 95%CI = 0.98–1.53).

### Stratified Analysis by Type of Disease

**Figure 2:**
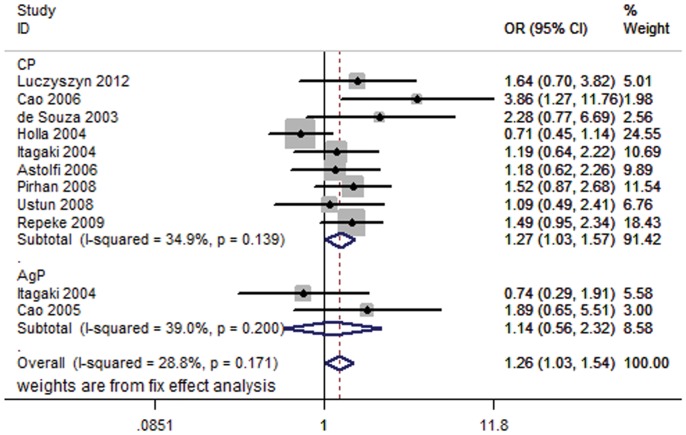
Forest plot of periodontitis risk associated with *MMP-1 g.-1607dupG* by type of disease under dominant model (2G/2G+1G/2G vs. 1G/1G). Fixed-effects model was used.

Appreciable differences were identified in the etiology feature between CP and AgP [33], implicating that there might be different genetic mechanism between them. Subgroup analysis showed that individuals were more susceptible to CP (OR = 1.27, 95% CI = 1.03–1.57) rather than AgP (OR = 1.14, 95% CI = 0.56–2.32) under the dominant model (Figure 2). The significantly elevated risk of CP was also observed in heterozygote comparison (OR = 2.01, 95% CI = 1.58–2.56) (Table 2).

**Table 2:** Meta-analysis for *MMP-1 g.-1607dupG* polymorphism and periodontitis risk.

Variables	N^a^	2G/2G versus1G/1G		2G/1G versus1G/1G		2G/2G+2G/1Gversus 1G/1G		2G/2G versus2G/1G+1G/1G		2G versus 1G	
		OR (95%CI)	*P* b	OR (95%CI)	*P* b	OR (95%CI)	*P* b	OR (95%CI)	*P* b	OR (95%CI)	*P* b
Total	10	**1.50** **(1.02–2.20)**	0.023	1.23(0.98–1.53)	0.522	**1.28** **(1.04–1.57)**	0.162	1.31(0.95–1.79)	0.012	**1.28** **(1.03–1.59)**	0.040
Type of disease											
CP	9	1.41(0.96–2.07)	0.040	**2.01** **(1.58–2.56)**	0.368	**1.27** **(1.03–1.57)**	0.139	1.17(0.96–1.43)	0.059	1.23(0.99–1.51)	0.012
AgP	2	1.54(0.28–8.55)	0.031	1.30(0.59–2.84)	0.222	1.14(0.56–2.32)	0.200	1.64(0.35–7.70)	0.008	1.34(0.48–3.73)	0.010
Severity of CP											
moderate	4	1.31(0.74–2.32)	0.312	1.25(0.73–2.16)	0.531	1.29(0.77–2.15)	0.409	1.14(0.76–1.70)	0.559	1.14(0.87–1.51)	0.306
Severe	5	**2.15** **(1.35–3.43)**	0.191	1.35(0.89–2.05)	0.753	**1.64** **(1.12–2.39)**	0.444	**1.86** **(1.31–2.64)**	0.187	**1.38** **(1.16–1.65)**	0.101
Ethnicity^c^											
Caucasian	4	1.22(0.59–2.51)	0.047	1.06(0.75–1.48)	0.363	1.06(0.78–1.45)	0.099	1.01(0.71–1.44)	0.160	1.04(0.86–1.25)	0.058
Asian	2	2.79(0.56–13.98)	0.020	1.31(0.74–2.32)	0.242	1.61(0.95–2.73)	0.071	2.01(0.72–5.61)	0.027	1.75(0.77–3.95)	0.011
Mixed	3	1.33(0.89–1.98)	0.976	**1.48** **(1.03–2.14)**	0.474	**1.42** **(1.01–1.99)**	0.793	1.05(0.77–1.45)	0.472	1.20(0.95–1.50)	0.094
Smoking status^c^											
Smoking	3	1.14(0.43–3.02)	0.222	1.12(0.54–2.34)	0.976	1.12(0.55–2.29)	0.682	1.19(0.53–2.65)	0.140	1.19(0.81–1.76)	0.415
non–smoking	7	1.36(0.80–2.32)	0.012	1.17(0.89–1.54)	0.497	1.25(0.84–1.87)	0.046	1.20(0.96–1.50)	0.014	1.21(0.90–1.65)	0.001

a Number of comparisons.

b P value of Q-test for heterogeneity test. Random-effects model was used when *P* value for heterogeneity test <0.05; otherwise, fix-effects model was used.

c AgP individuals are not included.

### Stratified Analysis by Severity

As showed in Table 2, significant association between the variation and severe CP was found in almost all types of comparisons. However, no significant relationship was found in moderate periodontitis (Table 2).

### Stratified Analysis by Ethnicity and Smoking Status

No significant association was found in Asians or Caucasians under any genetic model when analyzing the association between *MMP-1 g.-1607dupG* and CP by ethnicity. Similar effects were also observed in stratified analysis by smoking status.

### Heterogeneity Analysis

Heterogeneity among studies was observed in *MMP-1 g.-1607dupG* recessive model (I^2^ = 57.3%, *P = *0.012) and homozygote comparison (I^2^ = 53.3%, *P = *0.023). Z test of subgroup analysis indicated that type of periodontitis (*P*_z = _0.024) and smoking condition (*P*_z = _0.048) may be the main source of heterogeneity by homozygote comparison.

### Sensitive Analysis

**Figure 3:**
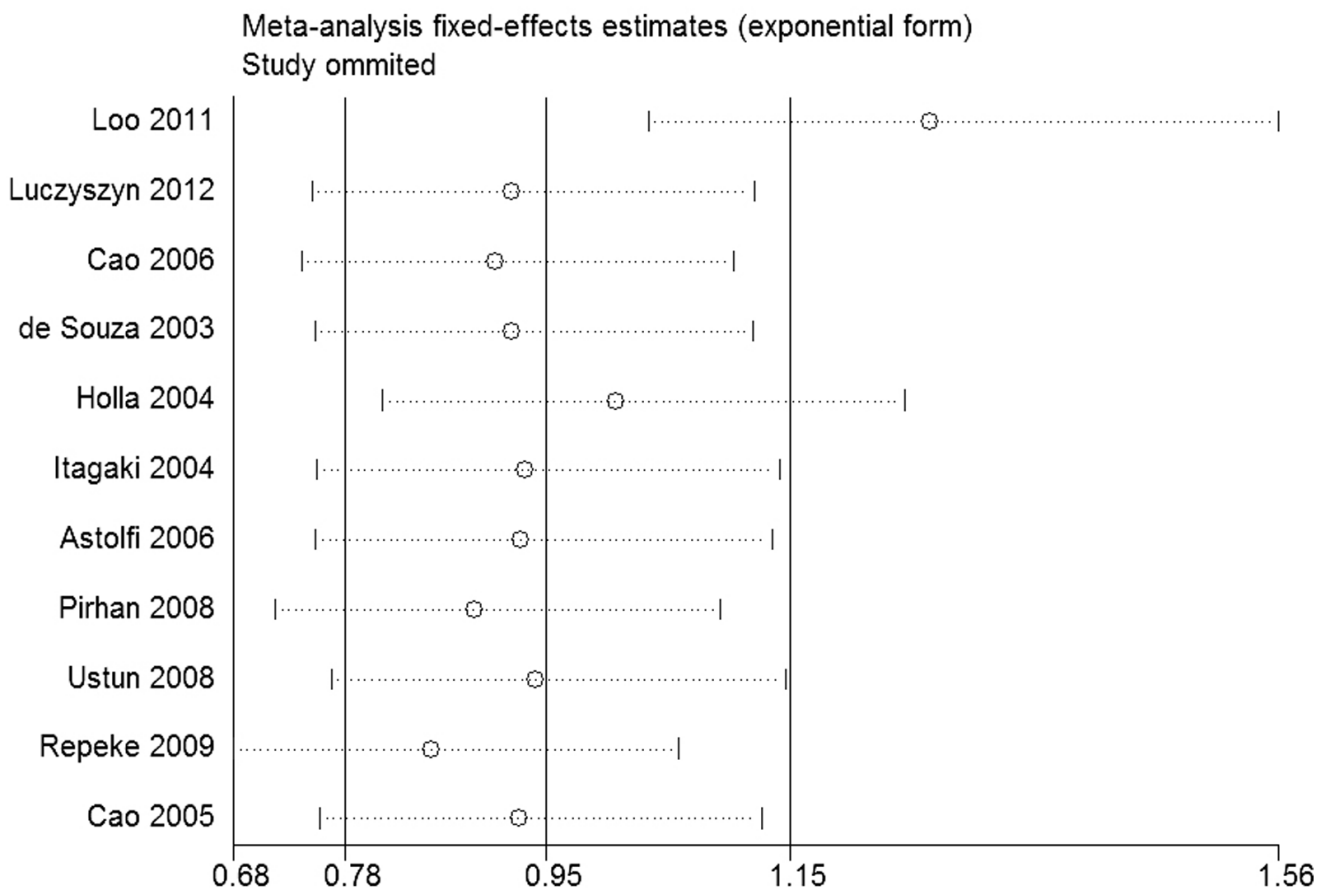
The result of sensitive analysis under the dominant model (2G/2G+1G/2G vs. 1G/1G). Fix effect model was used.

There are three of the 11 studies at variance with Hardy-Weinberg Equilibrium [28], [30], [31]. Among them, the study of Loo et al. [31] was found to be the major source of heterogeneity by sensitive analysis (Figure 3), and heterogeneity was effectively removed after excluding this study under the dominant model (I^2^ = 30.9%, *P = *0.162). In addition, the pooled ORs and 95% CI under the dominant model in overall comparison was obviously influenced (OR = 1.28, 95% CI = 1.04–1.57). (Before excluding, OR = 1.10, 95% CI = 0.60–2.03). The other two studies were reserved because they did not substantially affect the heterogeneity and the results (Figure 3). Then the statistical process was limited to ten articles left.

### Publication Bias Analysis

Egger’s test was conducted to assess the publication bias of the included studies. No publication bias was found in any genetic models. (t = −1.46, *P = *0.183 for 2G/2G+2G/1G versus 1G/1G).

## Discussion

Since 2003, there have been 11 studies focusing on the relationship between the SNP at position -1607 of *MMP-1* and periodontitis susceptibility. De Souza et al. found that subjects carrying -1607 2G allele tended to be more susceptible to severe CP in non-smoking Brazilian subjects [22]. Similar results were then observed in Czech [23] and Chinese subjects [26]. Elevated risk of CP and AgP was also found in 2G carriers of Asian origins by Loo [31] and Cao [25] et al. The other studies, however, obtained negative results [24], [27], [28]–[30], [32]. In sum, the definite role of *MMP-1 g.-1607dupG* in the development of periodontitis remains controversy.

By recruiting all of the above studies, we conducted the current meta-analysis and finally found that this variant could contribute to increased risk of periodontitis. In stratified analysis by type of disease, its association with susceptibility of CP rather than AgP was observed. Furthermore, its association with elevated risk of severe CP was also obtained. However, we did not find any meaningful associations in stratified analysis by ethnic and smoking status, both of which were considered to be the relevant factors of periodontitis.

MMP-1, playing a crucial role in paradentium destruction, was suggested to be an important risk factor of CP. It was previously demonstrated that 2G allele instead of 1G allel at *MMP-1* -1607 created a new 5′-GGA-3′ core recognition sequence for members of the erythroblast transformation specific family as the binding site, causing increased transcriptional activity, systemically accelerate *MMP-1* gene transcription and protein over-expression, expounding the molecular basis of a anabatic matrix degradation. [34]. In addition, some scholars demonstrated that *MMP1*-1607 2G allele was associated with increased MMP-1 mRNA expression in vivo [30]. Therefore, it was biologically plausible that individuals carrying *MMP1*-1607 2G allele were associated with over-expression of MMP-1, consequently contributing to more susceptibility to CP.

On the other hand, lack of association with AgP was found in the current study, not only implicating that MMP-1 might not been entirely activated in the pathogenesis of AgP, but also providing further evidence that AgP was different from CP in some aspects. Some scholars considered that AgP and CP shared some susceptibility genes, but not all [35]. Similarly, effect of some genetic variants had also been proved to be different [36] or even contract [37] on CP and AgP. Therefore, the role MMP-1 played in the development of AgP, if any, may not be as important as it did in CP. However, the negative association should be carefully interpreted because of the limited sample size.

Several limitations should be addressed. Firstly, the sample size is still a formidable problem. Based on the current sample size, we only had 63% power at a 0.05 or smaller with level to detect an OR of 1.2 or greater and 0.83 or smaller with an exposure frequency of 30%. Secondly, gene-environment interactions which may modulate the periodontitis susceptibility were limited owing to the lack of the origin data in the including studies.

Stated thus, our meta-analysis suggested that *MMP-1 g.-1607dupG* contribute to the elevated risk of CP. Further studies with large sample size and detailed information are needed to validate these results.

## Supporting Information

Checklist S1 **PRISMA 2009 checklist.**
(DOC)

Diagram S1 **PRISMA 2009 Flow Diagram.**
(DOC)
