# Supplementary material for: Association between MMP-1 g.-1607dupG Polymorphism and Periodontitis Susceptibility: A Meta-Analysis
Source: PLoS One. 2013 Mar 20;8(3):e59513. doi: 10.1371/journal.pone.0059513 (PMC3603913; doi:10.1371/journal.pone.0059513)
Supplement: Diagram S1 — PRISMA 2009 Flow Diagram. (DOC) [file pone.0059513.s002.doc]

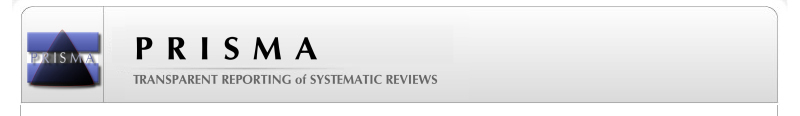
**PRISMA 2009 Flow Diagram**

**Screening**

**Included**

**Eligibility**

**Identification**

Records identified from Web of Science(n=18), records identified through PubMed searching (n=12)

Additional records identified through other sources
(n = 0)

Records after duplicates removed
(n = 19)

Records screened
(n = 19)

Unmatched titles and abstracts (n=8),
(n = 8)

Full-text articles assessed for eligibility
(n = 11)

Full-text articles excluded, with reasons
(n = 0)

Studies included in qualitative synthesis
(n =11)

Studies included in quantitative synthesis (meta-analysis)
(n = 10)
